# Attitude towards Telemonitoring in Orthodontists and Orthodontic Patients

**DOI:** 10.3390/dj9050047

**Published:** 2021-04-22

**Authors:** Domenico Dalessandri, Linda Sangalli, Ingrid Tonni, Laura Laffranchi, Stefano Bonetti, Luca Visconti, Alberto Signoroni, Corrado Paganelli

**Affiliations:** 1 Department of Medical and Surgical Specialties, School of Dentistry, Radiological Sciences, and Public Health, University of Brescia, Piazzale Spedali Civili 1, 25123 Brescia, Italy; lindasangalli87@gmail.com (L.S.); ingrid.tonni@unibs.it (I.T.); laura.laffranchi@unibs.it (L.L.); s.bonetti63@gmail.com (S.B.); l.visco@libero.it (L.V.); corrado.paganelli@unibs.it (C.P.); 2 Department of Information Engineering, University of Brescia, Via Branze 38, 25123 Brescia, Italy; alberto.signoroni@unibs.it

**Keywords:** orthodontics, telemonitoring, smartphone

## Abstract

The purpose of this study was to analyze the attitude of dentists and patients towards the use of Dental Monitoring^TM^ (DM), an orthodontic telemonitoring software. Thus, two different specially prepared specific questionnaires were administered to 80 dentists (40 were general dentists and 40 orthodontists) and 80 orthodontic patients. All dentists judged positively telemonitoring, as 96.25% of them considered telemonitoring indicative of high tech and high-quality treatment; 100% considered it a way to reduce the number of in-office visits; 17.5% agreed on a weekly telemonitoring frequency, 40% on a biweekly, and 42.5% on a lower frequency. Further, 97.5% of patients judged positively telemonitoring; 81.25% of them considered telemonitoring indicative of high-tech treatment; 81.25% declared to be interested in reducing the number of in-office visits through telemonitoring; 27.5% agreed on taking self-picture every week, 57.5% every two weeks, and 15% on a lower frequency. Both patients and dentists positively judged telemonitoring, considering it a technologically advanced tool increasing the perception of quality and accuracy of the treatment. Both groups were interested in reducing the number of in-office visits, although not all of them revealed to be ready to invest more money and time in it.

## 1. Introduction

Orthodontic therapies can be often long-lasting treatments, particularly when undesired side effects occur and they are recognized by the clinician only after several months [1].

In the last few years, several approaches have been suggested to reduce orthodontic treatment duration, ranging from surgical and non-surgical techniques. These approaches have the aim to speed up the biological mechanisms involved in tooth movement, to individualized orthodontic appliances fabrication and to optimize efficient clinical protocols elaboration, trying to strictly control tooth displacement direction and avoid time-wasting round trip movements [2,3,4,5,6,7]. Regardless of the strategy applied, a strict control of treatment progression is of crucial importance in order to save time and avoid unexpected complications, or at least minimize their consequences [8].

Recently an increased diffusion and technological advances in computers, smartphones and tablets, especially among adolescents, introduced new communication habits and a more demanding attitude towards immediate information need and assistance request satisfaction [9]. The medical and dental world were also involved in this “permanently online” revolution. The Association of American Medical Colleges named it teledentistry, which is “the use of telecommunications technology to send data, graphics, audio, and video images between participants who are physically separated (i.e., at a distance from one another) for the purpose of clinical care” [10]. It includes all the ways to share digital information through communication technology [11]: not only web browsing and distance learning, but also development of thousands of different apps dedicated to healthcare related educational content delivery, medical data collection and patient monitoring and motivation [12,13,14].

The benefits of teledentistry are many, including strict remote monitoring of the evolution of the treatment, increased access to oral care, better patient education, earlier diagnosis of cavities, increase in patient collaboration, reduction of the need for travelling by patients and orthodontists, constant control on appliances integrity and oral hygiene efficacy, elimination of unnecessary appointments, reduction of side-effects by alerting the clinician in case of changing conditions as soon as they occur, and a reduction of in-office check-ups and of emergencies [15,16].

Innovative technologies, such as kiosks, website monitoring applications, mobile wearable devices and video conferencing are listed among teledentistry opportunities [17]. Considering that the majority of the population owns a smartphone [18], several studies have already confirmed the use of different text messaging apps, such as WhatsApp© (WhatsApp© Inc., Mountain View, CA, USA), Telegram© (Telegram Messenger Inc., London, UK) or WeChat© (Tencent Holdings Limited, Shenzhen, China), as teledentistry opportunities [19,20] especially when patients want to communicate with clinicians for emergencies [21] or to promote a good oral health behavior [22,23,24]. 

A step forward was made by Dental Monitoring^TM^ (DM, Dental Monitoring©, Montreal, France) [25], a software system that allows the therapist to maintain control over teeth movement, appliance integrity, and oral hygiene efficacy during orthodontic treatment through the analysis of pictures periodically taken by the patient. It consists of three integrated platforms: a mobile app for the patient, a patented teeth movement-tracking algorithm, and a web-based Doctor Dashboard^®^ where the clinician can check the patient’s progress, tooth by tooth, in terms of dental angulation, inclination, and intrusion/extrusion. DM receives the patient’s pretreatment photographs and a 3D model in stereolithography file format (.stl). Every time the patient takes intramural pictures, they are automatically uploaded to cloud-based servers. DM’s algorithms link the picture with the 3D model and calculate baseline tooth positions, overjet, overbite, and interarch relationships, by creating a multidimensional information map (IM) of the teeth. The results are uploaded to the Doctor Dashboard^®^ in the form of graphs, photos, and 3D models. 

Considering that medical telemonitoring relies on the active involvement of both the patient and the clinician, the aim of this study was to analyze orthodontists’ and patients’ attitude towards the use of Dental Monitoring^TM^, through the filling of a questionnaire. 

## 2. Materials and Methods 

Eighty dentists (25–60 years old) and eighty patients (12–50 years old) of both genders, undergoing an orthodontic treatment with clear aligners or buccal multibrackets appliances, were enrolled in this multicenter university and private practice-based cross-sectional study. Half of the dentists were orthodontists; the other half were general dentists with a special interest in orthodontics. Two different questionnaires were used to evaluate doctors’ perceptions and patients’ perceptions, investigating their attitudes towards telemonitoring and their opinion regarding its usefulness.

We conducted a literature review to identify if previously validated questionnaires already existed, without success. We than decided to construct two brief, specific questionnaires based on experts’ judgment, the pertaining literature review and patients’ and doctors’ interviews, using simple and short items assessing single issue. A preliminary pilot test was performed on a group of ten orthodontic patients and ten doctors, in order to identify any possible source of confusion about any items and to test the validity and reliability of the questionnaires; after that, the two questionnaires were consequently revised.

The questionnaire for the doctors consisted of closed-ended items on demographic data and investigated their willingness to adopt the above-mentioned software system and the fees they would be willing to charge and pay, in order to increase the accuracy of their treatment. The questionnaire for the patients included close-ended questions on demographic data, namely on their willingness to actively participate in the orthodontic treatment and to pay an extra fee, in order to reduce treatment length and increase treatment accuracy. The inclusion criteria were the following: the dentists had to be between an age of 25–60 years old and practice orthodontics. As for the patients, they had to be currently in orthodontic treatment with clear aligners or buccal multi brace system. Exclusion criteria were severe medical history, patients less than 12 years of age and craniofacial deformities. The questions were answered individually at office. 

All subjects gave their informed consent for inclusion before participating in the study. According to the guidelines of our internal ethical committee, the study protocol was not submitted for specific approval since no clinical intervention was performed on patients and the questionnaires submitted to the subjects involved in this survey were anonymous. The study was conducted in accordance with the Declaration of Helsinki.

Once patients and orthodontists declared their willingness to participate, an oral presentation explaining the use of Dental Monitoring^TM^ system through demonstrative videos and printed leaflets was shown to the participants. After that, patients and orthodontists attended practical training sessions focused on how to use the DM system, and finally they were allowed to test the software on their own for one month. The study coordinator was available to answer any questions while the participant completed the questionnaire. Once the participants had completed it, each questionnaire was put in a closed box to keep it completely anonymous.

All subjects gave their informed consent for inclusion before they participated in the study. The study was conducted in accordance with the Declaration of Helsinki, and the protocol was approved by the Ethics Committee of Strengthening the Reporting of Cross-Sectional Studies (281322DM01, 5 May 2021).

### Statistical Analysis

Descriptive statistics were used to summarize the data. Frequency distributions were used for categorical variables and means, standard deviations, and percentile distributions were used for continuous data. Sociodemographic characteristics of both doctors and patients were examined. These characteristics included sex, highest level of education, job, age, country of residence, smartphone ownership, habit of being online daily, and awareness of telemonitoring systems.

Sociodemographic data were recorded and chi-square contingency tables were utilized to analyze the effect of sex, age and smartphone ownership on telemonitoring attitude. A multiple regression analysis was used to estimate this effect. When common questions were present in both questionnaires, a comparison was made between patients’ and orthodontists’ attitudes.

Responses of the two surveys (practicing orthodontists and active patients) were tabulated by the primary variables of interest. Chi-square tests were used to examine the association between gender and willingness to pay additional fee by orthodontists and patients, the association between age and availability to take pictures every two-weeks or weekly by orthodontists and patients, the association between age and interest in reducing in-office visits by the patients. 

All tests were 2-sided and a *p* value of <0.05 was deemed to be statistically significant. All data were recorded into an Excel data sheet. All analyses were conducted using Excel spreadsheets (version 15.5; Microsoft Excel, Microsoft, Redmond, WA, USA). 

## 3. Results

The survey was administered from October to December 2019. The sociodemographic characteristics of the doctors are summarized in Supplementary Table S1. The sample was composed of 80 dentists (45 females and 35 males), with 46 of them aged between 25 and 39 and 34 of them between 40 and 60 years old. All the doctors lived in Italy. Regarding their highest level of education, 57.5% of them completed a post-graduate program in Orthodontics, 10% of them concluded a PhD, and 32.5% of them achieved a bachelor degree in Dentistry.

The sociodemographic characteristics of the patients are summarized in Table S2. The patients enrolled were 47 females and 33 males; 22 of them were under 14 years old; 21 of them were aged between 15 and 18; 20 of them were between 19 and 30; 11 of them had between 31 and 40. The remaining six were between 41 and 50 years old. 

Supplementary Tables S3 and S4 show the other questions submitted to doctors and patients, respectively.

Analysis of the socio-demographic data reveals that gender and age (Supplementary Table S5 and Table S6 respectively) did not influence any attitude towards telemonitoring among patients (*p* value = 0.73 and *p* value = 0.81, respectively). As far as dentists are concerned, all of them judged positively telemonitoring, regardless of age and gender.

On the contrary, smartphone ownership was significantly associated with a positive judgement. Indeed, 79 dentists owned a smartphone and declared to be online daily; only 1 of them didn’t own a smartphone, therefore this subject did not appear to be online daily. As for the patients, all of them declared to own a smartphone, and only 3 of them claimed not to be online daily. 

Regarding the awareness about the possibility to use a smartphone for telemonitoring, the higher the level of education of the participants, the more they knew about telemonitoring; indeed, 53.75% of the orthodontists were already aware of it, whereas 58.75% of the patients did not know about the possibility.

After the oral presentation and the practical training session, all 80 dentists judged this new system of long-distance monitoring positively. Almost the totality of them (96.25%) considered telemonitoring indicative of high-tech and high-quality treatment; all of them declared that telemonitoring system can reduce the number of in-office visits. 23 dentists have already used a smartphone to monitor their patients: 15 dentists monitored 0–25% of the patients, six of them supervised 25–50% of the patients, and two used a remote-control system on 50–75% of the patients. The purposes were several: emergency reason (16 of them), oral hygiene check (one of them), compliance on wearing external devices (seven of them), control of treatment progress (three of them). Conversely, one of the orthodontists declared useless to periodically measure teeth movements.

A discrepancy in the answers was found when asked how often they would be willing to examine patients’ pictures: 46 of them declared they were willing to review the photographs every two weeks; of these doctors, 14 would have been willing to check them every week. Moreover, 50 orthodontists were concerned about the economic impact on their financial budget.

As for the patients, most of them found the DM software easy to understand. Only two of them did not judge telemonitoring positively. Meanwhile, 65 of them (81.25%) declared to be interested in reducing the number of in-office visits, 65 of them (81.25%) considered the use of telemonitoring a high-tech marker, and 49 of them (81.7%) considered it indicative of high-quality treatment. Different answers emerged when the availability to take pictures of themselves was investigated: 68 (85%) of them declared to be willing to take pictures every two weeks, but only 22 of them (27.5%) assented to perform it weekly. Lastly, 52 of them (65%) did not agree to pay an additional fee in order to add a telemonitoring system during their orthodontic treatments.

When questioned regarding their concerns about an additional fee, male dentists seemed more willing to be charged; indeed, as Supplementary Table S7 displays, 48.6% of them declared not to be worried about the economic impact on their financial budget, compared to 35.6% of female dentists. There was no significant difference in the answer whether the doctor was an orthodontist or a clinician with interest in orthodontics.

As for the patients, the situation between females and males was similar: the 36% of the male patients and the 28.5% of the female patients accepted to be charged an additional fee in order to benefit from this service. Supplementary Tables S8 and S9 show the results of the performed chi-square tests, revealing that the correlation between gender and concerns about an additional fee among either the patients and the doctors was not statistically significant (*p* = 0.54 and *p* = 0.26 respectively).

A further analysis conducted was the association between patients’ age and their willingness to take pictures of their own teeth bi-weekly or weekly, and the association between orthodontists’ age and their willingness to analyze pictures bi-weekly or weekly (Supplementary Table S10).

Regarding the orthodontists, an equal number of doctors between 40 and 60 years old declared their availability to analyze pictures received from their patients every two weeks. Interestingly, none of them assented to invest time in examining the pictures every week. Different was the situation when considering the young doctors (25–39 years old): 22.2% of them accepted to investigate the photographs weekly.

The percentage of doctors that stated their availability in analyzing pictures every two weeks was quite similar: 58.7% (25–39 years old) compared to 52.9% (40–60 years old). Yet, the correlation between age and willingness to take pictures weekly among the orthodontists was not statistically significant (Table 1). When investigating the correlation between category (general dentist vs orthodontist) and willingness to commit bi-weekly or weekly to taking pictures, the difference was found not statistically significant (*p* = 0.17 and *p* = 0.56).

Regarding the answers given by the patients, they were divided into two categories, younger and older than 18 years old. Interestingly, the younger they are, the more willing they appear in taking pictures of their mouth. 93% of the patients under 18 years old accepted to invest time in taking photographs every two weeks, compared to the 81% of the patients over 18 years old. Moreover, a statistically significant difference was notable when considering the patients that accepted to commit weekly: 58% of the patients under 18 years old declared their availability in taking pictures every week, compared to 35% of the older (*p* value = 0.039), as shown in Table 2.

A further examination was conducted by analyzing the data collected among the patients, to investigate whether there was an association between age and interest in reducing the number of in-office visits, assuming that Dental Monitoring^TM^ can reduce the number of conventional chair-side check-ups (Table 3). 

The majority of the patients expressed their desire to decrease the visits, regardless their age (79% of the patients were under 18 years old and 86.4% of the patients were over 18 years old). As shown in Table 4, the chi-square *p* value shows a not statistically significant correlation between age and willingness to reduce the chair-side visits. 

## 4. Discussion

In general, teledentistry has an enormous potential to expand the oral healthcare being provided. This study was focused on apps developed for orthodontists, but it can be extended to any dental care professional who wishes to improve diagnostic care, to gain expert advice, to determine referrals, to strictly follow up the patients. Several quantitative systematic reviews demonstrate the effectiveness of teledentistry for various specialties, including endodontics, oral surgery, oral medicine, periodontics, prosthetics, pediatrics, conservative dentistry, and orofacial pain [26,27].

The method of the questionnaire to evaluate what the population of potential patients thinks about the tool in exam has often been used in the literature. For example, the “Health Effect and Readiness” Questionnaire was utilized for diabetic patients, to check its reliability and validity [28]. Gagnon MP et al. found positive responses by submitting a questionnaire, based on the technology acceptance model (TAM), evaluating the face and content validity of telemonitoring system, however, the questionnaires were distributed among nurses and doctors, not to patients [29]. Finkelstein J et al. analyzed the attitude that the patients have towards the technology, drawing the conclusions that the self-tests taken by the patients with no computer background are valid and comparable to those tests collected under the supervision of a trained medical professional [30]. In their study, Aamodt et al. sent questionnaires to physician and nurses, not to patients, describing their perception on the fact that telemonitoring could increase the quality of care for heart failure patients [31]. Ruiz-Lopez del Prado et al. applied the use of a questionnaire on a sample of patients to evaluate the state of oral health in preoperative anesthetic evaluations [32]. Uribe et al. used the method of the questionnaire to evaluate patients’, parents’ and orthodontists’ perspective on orthodontic treatment duration and techniques for accelerating the rate of tooth movement [33].

Compared to the studies found in the literature, the current investigation showed the attitude of both orthodontists and orthodontic patients towards a long-distance monitoring tool as Dental Monitoring^TM^. Moreover, the sample of the doctors was divided into two categories: doctors with a special interest in orthodontics and doctors who provides only orthodontic treatments to their patients.

When analyzing the answers given by the two groups among the doctors, no statistically significant differences were found. In general, some of the doctors were already aware of the possibility to monitor their patient by telemonitoring system and more than half of them already used it, with several purposes. Overall, many clinicians are already engaging in teledentistry. Educational orthodontic blogs, Facebook^®^ forums (Facebook Inc., Menlo Park, CA, USA), and interactive web-based coaching are few examples of the common use of teledentistry among the practitioners. The orthodontists, which were not aware yet, showed a good attitude towards it, but just few of them revealed their openness to start checking patients frequently. In particular, very few accepted to examine patients’ pictures weekly. Likely, this frequent checking requires an important effort and availability of time by the doctors. Furthermore, traditionally the clinician is more inclined to check and relate to his patients in person; this can also explain why some of the orthodontists replied negatively. Next, some of them underlined their worry about the economic impact of the telemonitoring, a further possible explanation of their reticence. 

On the patient side, a majority of them were not aware of the possibility of telemonitoring, confirming the results of a study by Sharif et al. [34]. Some of them declared that the use of telemonitoring was not indicative of high tech and high-quality treatment. This negative statement is probably explained by the fact that they do not judge a high-quality treatment on the base of how many technologies their orthodontist can offer them. Even a conventional treatment appears to be a high level one if it leads to good results. Furthermore, the opinion that telemonitoring was not indicative of high tech could mean that the potential for dental calculation with this app was underestimated. 

When investigated their availability to take pictures of themselves, the younger they were, the more willing they appeared. Nevertheless, just very few of them accepted to a weekly commitment. The same data was underlined by the answers given by the orthodontists: a weekly request seems too demanding and rigorous to both of them. In that case, they seem to prefer the traditional monthly check. 

For the reason of time saving and high tech and high-quality treatment, some of the patients stated their willingness to pay an additional fee to benefit from telemonitoring system for their orthodontic treatment.

Moreover, the majority of them were interested in reducing the number of in-office visits; in particular, the older they were, the more a reduction in the number of scheduled appointments would be appreciated. Further, the association between age and interest in reducing the in-office visits did not appear to be statistically significant. Few patients were not interested in reducing the number of chair-side appointments. A reason of this statement could be based on the high-value given to doctor-patient relationship. Technological advances come with a dark side as well [35]. A doctor–patient relationship is based on a set of parameters which include the interpersonal relationship between the patient and the doctor. Based on the primary care assessment survey model, measures of the interpersonal relationship are associated with communication, interpersonal care, contextual knowledge of the patient, and trust [36]. Several studies report the importance of a face-to-face rapport, preferring a real consultation over an exclusive use of telemonitoring technology [37]. The key for the future of applying remote control system would be in balancing the benefits of these advances in technologies and direct patient-doctor relationships. An excellent standard of care can be obtained with the convenience and reduced costs of remote monitoring while maintaining an individual patient-to-patient bases relationship [38].

In summary, the strengths of this research are: the use of two specifically developed questionnaires, both with a high validity and reliability, which could be used in the future to perform similar studies also by other research groups; direct involvement of patients, who are the final beneficiaries of telemonitoring systems; and the focus on a software specifically conceived for orthodontic use. On the other hand, the main limitations of this research are the absence of an objective evaluation of patients’ and doctors’ adherence in real life to a telemonitored orthodontic treatment approach and the absence of the evaluation of the impact of the use of this kind of telemonitoring software on parameters such as patients’ satisfaction, compliance during both active treatment and retention phase, oral hygiene maintenance, treatment duration, costs, and quality. Future researches on telemonitoring software should therefore focus on clinically relevant endpoints, such as the impact on treatment duration and results of a high compliance-demanding and increasingly popular type of orthodontic appliance, such as that with clear aligners, the influence on patient’s quality of life, and the satisfaction with regard to treatment management and results, or the usefulness in daily oral hygiene maintenance and management of sudden emergencies [39,40,41,42,43,44,45,46,47,48].

## 5. Conclusions

The patients in this study showed a more positive attitude towards telemonitoring compared to the doctors. Both patients and doctors judged telemonitoring positively, considering it a technologically advanced tool able to increase the perception of quality and accuracy of the treatment. Patients and doctors seem to be interested in reducing the number of in-office visits, but not all of them revealed to be ready to invest more money and time in it.

## Figures and Tables

**Table 1:** Chi-square tests performed among the orthodontists, investigating the correlation between age and availability to take/examine pictures bi-weekly or weekly, respectively.

Doctors	25–39	40–60	Dentists	Orthodontists
**Yes**	27	18	26	20
**No**	29	16	14	20
Bi-weekly *p*-value	0.61		0.17	
**Doctors**	**25–39**	**40–60**	**Dentists**	**Orthodontists**
**Yes**	6	8	6	8
**No**	34	32	34	32
Weekly *p*-value	0.26		0.56	

**Table 2:** Chi-square tests performed among the patients, investigating the correlation between age and availability to take/examine pictures twice-a-week or weekly, respectively.

Patients	<18	>18
Yes	40	30
No	3	7
Bi-weekly *p*-value	0.11	
Patients	<18	>18
Yes	25	13
No	18	24
Bi-weekly *p*-value	0.04	

**Table 3:** Association between age and interest in reducing the number of in-office visits among the patients.

Question	Response	Patients	
		<18 Years Old	>18 Years Old
Are you interested in reducing the number of in-office visits?	Yes	34 (79%)	32 (86.40%)
	No	9 (21%)	5 (13.60%)

**Table 4:** Chi-square test examining the association between age and interest in reducing the number of in-office visits among the patients.

Patients	<18	>18
Yes	34	32
No	9	5
*p*-value	0.38	

## Data Availability

The data supporting the findings of the article is available in the University of Brescia repository “OPENBS IRIS” at http://hdl.handle.net/11379/543696 (accessed on 1 April 2021), reference number 543696 (Attitude towards Telemonitoring in Orthodontists and Orthodontic Patients database).

## Supplementary Materials

The following are available online at https://www.mdpi.com/article/10.3390/dj9050047/s1, Table S1: Sociodemographic data of the doctors participating in the survey, Table S2: Sociodemographic data of the patients participating in the survey, Table S3: Questionnaire for doctors, Table S4: Questionnaire for patients, Table S5: Association between gender and attitude toward telemonitoring among the patients, Table S6: Association between age and attitude toward telemonitoring among the patients, Table S7: Association between gender and concerns about an additional fee, Table S8: Chi-square test investigating the correlation between gender and concerns about an additional fee among the patients, Table S9: Chi-square test investigating the correlation between gender and concerns about an additional fee among the doctors, Table S10: Association between age and availability to take / examine pictures bi-weekly or weekly.
