# Supplementary material for: Attitude towards Telemonitoring in Orthodontists and Orthodontic Patients
Source: Dent J (Basel). 2021 Apr 22;9(5):47. doi: 10.3390/dj9050047 (PMC8143570; doi:10.3390/dj9050047)
Supplement: Supplementary file 1 [file dentistry-09-00047-s001.zip › dentistry-888443-supplementary.pdf]

Characteristic	Response	n (%)
Gender	Male	35 (43.75%)
	Female	45 (56.25%)
Age	25-39	46 (57.50%)
	40-60	34 (42.50%)
Job	Orthodontist	40 (50%)
	Dentist	40 (50%)
Country of residence	Italy	80 (100%)
	Foreign country	0 (0%)
Level of education	Bachelor Degree	26 (32.50%)
	Post-graduate	46 (57.50%)
	PhD	8 (10%)

Table S1. Sociodemographic data of the doctors participating in the survey.

Characteristic	Response	n (%)
Gender	Male	33 (41.25%)
	Female	47 (58.75%)
Age	12-14	22 (27.50%)
	15-18	21 (26,25%)
	19-30	20 (25%)
	31-40	11 (13.75%)
	41-50	6 (7.50%)
Job	Student	45 (56.25%)
	Lawyer	3 (3.75%)
	Teacher	10 (12.50%)
	Medical field	8 (10%)
	Others	14 (17.50%)
Country of residence	Italy	80 (100%)
	Foreign country	0 (0%)
Level of education	Secondary school	47 (58.75%)
	High school	12 (15%)
	Bachelor Degree	21 (26.25%)

Table S2. Sociodemographic data of the patients participating in the survey.

Characteristic	Response	n (%)
Do you own a smartphone?	Yes	79 (98.75%)
	No	1 (1.25%)
Are you online daily?	Yes	79 (98.75%)

	No	1 (1.25%)
Are you aware of the possibility to use a smartphone for telemonitoring?	Yes	43 (53.75%)
	No	37 (46.25%)
Do you positively judge telemonitoring?	Yes	80 (100%)
	No	0 (0%)
Have you ever used a smartphone-based monitoring system?	Yes	23 (28.75%)
	0-25%	15 (18.75%)
	25-50%	6 (7.50%)
	50-75%	2 (2.50%)
	75-100%	0
	No	57 (71.25%)
If yes, for which purpose?	Emergencies	16 (20%)
	Hygiene collaboration	1 (1.25%)
	wearing external devices	7 (8.75%)
	treatment progress	3 (3.75%)
Do you think telemonitoring can reduce the number of in-office visit?	Yes	80 (100%)
	No	0 (0%)
Do you consider telemonitoring indicative of high-tech and high-quality?	Yes	77 (96.25%)
	No	3 (3.75%)
Do you consider useful to periodically measure teeth movements?	Yes	79 (96.50%)
	No	1 (2.50%)
Would you be willing to examine patients pictures every 2 weeks?	Yes	46 (57.50%)
	No	34 (42.50%)
Would you be willing to examine patients pictures every week?	Yes	14 (17.50%)
	No	66 (82.50%)
Are you concerned about the economic impact on your financial budget?	Yes	55 (68.75%)
	No	25 (31.25%)

Table S3. Questionnaire for doctors.

Question	Response	N
Do you own a smartphone?	Yes	80 (100%)
	No	0 (0%)
Are you online daily?	Yes	77 (96.25%)
	No	3 (3.75%)
Are you aware of the possibility to use a smartphone for telemonitoring?	Yes	33 (41.25%)
	No	47 (58.75%)
Do you positively judge telemonitoring?	Yes	75 (93.75%)
	No	5 (6.25%)
Are you interested in reducing the number of in-office visits?	Yes	65 (81.25%)
	No	15 (18.75%)
Do you consider the use of telemonitoring indicative of high-tech treatment?	Yes	65 (81.25%)

	No	15 (18.75%)
Do you consider the use of telemonitoring indicative of high-quality treatment?	Yes	65 (81.25%)
	No	15 (18.75%)
Would you be willing to take pictures of your teeth every 2 weeks?	Yes	68 (85%)
	No	12 (15%)
Would you be willing to take pictures of your teeth every week?	Yes	22 (27.50%)
	No	58 (72.50%)
Are you willing to pay an additional fee to use a telemonitoring system during your treatment?	Yes	28 (35%)
	No	52 (65%)

Table S4. Questionnaire for patients.

Patients	Male	Female
**Yes**	31	44
**No**	2	3
p-value	.73	

Table S5. Association between gender and attitude toward telemonitoring among the patients.

Patients	<18	>18
**Yes**	41	35
**No**	2	2
p-value	.81	

Table S6. Association between age and attitude toward telemonitoring among the patients.

Question	Response	Patients		Doctors		Dentists	Orthodontists
		males	females	males	females		
Are you concerned about the economic impact on your financial budget?	Yes	22 (67%)	33 (72.22%)	18 (51.40%)	29 (64.40%)	24 (60%)	24 (60%)
	No	11 (33%)	14 (29.78%)	17 (48.60%)	16 (35.60%)	16 (40%)	16 (40%)

Table S7. Association between gender and concerns about an additional fee.

Patients	Male	Female
**Yes**	22	33
**No**	11	14
p-value	.73	

Table S8. Chi-square test investigating the correlation between gender and concerns about an additional fee among the patients.

Doctors	Male	Female
**Yes**	18	29
**No**	17	16
p-value	.24	

Table S9. Chi-square test investigating the correlation between gender and concerns about an additional fee among the doctors.

Question	Response	Patients		Dentists	
		**< 18 years old**	**> 18 years old**	**25-39 years old**	**40-60 years old**
Would you be willing to take / analyse pictures of the teeth every 2 weeks?	Yes	40 (93%)	30 (81%)	27 (58.70%)	18 (52.90%)
	No	3 (7%)	7 (19%)	19 (41.30%)	16 (47.10%)
Would you be willing to take / analyse pictures of the teeth every week?	Yes	25 (58%)	13 (35%)	6 (22.20%)	1 (7.70%)
	No	18 (42%)	24 (65%)	21 (77.80%)	12 (92.30%)

Table S10. Association between age and availability to take / examine pictures bi-weekly or weekly.
